# Assessment of Pre-Pregnancy Dietary Intake with a Food Frequency Questionnaire in Alberta Women

**DOI:** 10.3390/nu7085277

**Published:** 2015-07-27

**Authors:** Stephanie M. Ramage, Linda J. McCargar, Casey Berglund, Vicki Harber, Rhonda C. Bell, the APrON Study Team

**Affiliations:** 1 Department of Agricultural, Food and Nutritional Science, 4-002 Li Ka Shing Centre for Health Research Innovation, University of Alberta, Edmonton, AB T6G 2E1, Canada; 2 Faculty of Physical Education and Recreation, 3-100 University Hall, Van Vliet Complex, University of Alberta, Edmonton, AB T6G 2H9, Canada

**Keywords:** pre-pregnancy, dietary intake, diet assessment, food frequency questionnaire

## Abstract

Purpose: Pre-pregnancy is an under-examined and potentially important time to optimize dietary intake to support fetal growth and development as well as maternal health. The purpose of the study was to determine the extent to which dietary intake reported by non-pregnant women is similar to pre-pregnancy dietary intake reported by pregnant women using the same assessment tool. Methods: The self-administered, semi-quantitative food frequency questionnaire (FFQ) was adapted from the Canadian version of the Diet History Questionnaire, originally developed by the National Cancer Institute in the United States. Pregnant women (*n* = 98) completed the FFQ which assessed dietary intake for the year prior to pregnancy. Non-pregnant women (*n* = 103) completed the same FFQ which assessed dietary intake for the previous year. Energy, macronutrients, and key micronutrients: long-chain omega-3 fatty acids, folate, vitamin B_6_, vitamin B_12_, calcium, vitamin D and iron were examined. Results: Dietary intake between groups; reported with the FFQ; was similar except for saturated fat; *trans* fat; calcium; and alcohol. Pregnant women reported significantly higher intakes of saturated fat; *trans* fat; and calcium and lower intake of alcohol in the year prior to pregnancy compared to non-pregnant women who reported intake in the previous year. Conclusions: Despite limitations; a FFQ may be used to assist with retrospective assessment of pre-pregnancy dietary intake.

## 1. Introduction

It is well known that dietary intake during pregnancy impacts fetal growth and development as well as maternal health. However, a woman’s weight status and dietary intake over the course of her life and particularly before becoming pregnant may also affect fetal growth and development and long-term maternal health [1,2]. Maternal obesity prior to pregnancy has been associated with negative maternal outcomes including increased risk of miscarriage, gestational diabetes, pregnancy-induced hypertension, venous thrombo-embolism, induction of labour, caesarean delivery, excessive gestational weight gain, and post-partum weight retention [3,4,5,6]. Pre-pregnancy maternal obesity is also associated with increased risk of poor infant outcomes including fetal macrosomia, childhood obesity and increased risk of diabetes later in life [3,5,6]. Due to its importance for both maternal and infant outcomes, weight status of women prior to pregnancy has been emphasized as a key variable in the United States Institute of Medicine gestational weight gain guidelines published in 2009 [7].

While the effects of pre-pregnancy dietary intake are not well characterized, improvements prior to pregnancy may decrease the risk of poor maternal and fetal outcomes. Maternal nutrition status prior to conception and during the peri-implantation phase is believed to affect embryonic and fetal growth [8]. Recent research from the Nurses’ Health Study indicated that increased intake of fried foods prior to pregnancy was associated with increased risk of gestational diabetes even after adjusting for multiple factors including age, body mass index (BMI), family history of diabetes, total energy intake and diet quality [9]. Results from studies in animal models are consistent with these results in humans. For example, an animal model fed a high-fat diet prior to pregnancy demonstrated negative effects in offspring including increased insulin like growth factor 2 receptor mRNA, smaller offspring that undergo catch-up growth, and in males, increased cholesterol, increased percent body fat, and glucose intolerance in later life [10]. Another animal model that utilized a high-fat diet prior to pregnancy found the offspring had increased lipid droplet storage in hepatocytes and increased body weight compared to controls fed standard chow during pre-pregnancy as well as pregnancy and lactation [11]. Liver tissue in offspring also had a unique up-regulation of Acacb and Scd1 gene expression in those mice fed a high-fat diet during the pre-pregnancy period only [11].

In addition to high dietary fat intake, maternal nutrient deficiencies prior to pregnancy have also been linked with fetal developmental defects. The most commonly known defect related to pre-pregnancy nutrition is the increased risk of neural tube defects (NTDs) with low levels of maternal folate [12]. Other nutrient insufficiencies or deficiencies may also be important. One study found that after adjusting for energy intake, low maternal dietary intake of vegetable protein, fibre, beta-carotene, vitamin C, vitamin D, iron and magnesium were linked to an increased risk of orofacial clefts in newborns [13].

Determining an appropriate method of dietary intake assessment during the pre-pregnancy period is important to support future interventions. For example, it has been found that women who received pre-conception advice from a health professional were more likely than other women to make positive behavior changes prior to pregnancy including taking supplementary folic acid and consuming a healthier diet [2]. A pre-pregnancy dietary intake assessment tool that has the potential to track changes could assist in determining the impact of interventions in creating positive diet changes during this critical period. However, recruitment of women before pregnancy is difficult, thus a tool that examines dietary intake retrospectively would be important.

This study was completed as a pilot project to support an ongoing cohort study: Alberta Pregnancy Outcomes and Nutrition (APrON) [14]. The APrON study is exploring the relationship of maternal dietary intake and nutrient status during pregnancy with maternal mental health, birth outcomes and child neurodevelopment up to three years of age. Women were enrolled in the APrON study once they became pregnant, however, as described above, it was important to assess dietary intake prior to pregnancy. The purpose of the study was to determine the extent to which dietary intake reported by non-pregnant women is similar to pre-pregnancy dietary intake reported by pregnant women using the same assessment tool.

## 2. Methods

### 2.1. Participants

Pregnant and non-pregnant women were recruited from Edmonton, Alberta, Canada. For the pregnant group, any woman who was currently pregnant was eligible to participate in the study. There were no restrictions placed on number of weeks gestation. For the non-pregnant group, women who had not been pregnant during the past 12 months and were aged 17–45 years were eligible to participate. We have no information as to whether any of the participants were planning a pregnancy. Women in both groups were excluded if they were unable to speak, read, or write in English.

A sample size of 100 participants in each group was chosen in order to allow for comparison between groups; this sample size has been identified as appropriate for assessment of validity of FFQs [15]. Although this study did not directly assess validity of the FFQ, other aspects of the study beyond the scope of this short report did examine relative validity.

Different recruitment strategies were utilized for the pregnant and non-pregnant groups. Recruitment of pregnant participants was also completed as a pilot test for one of the APrON study recruitment strategies. This was organized in conjunction with the Women and Children’s Health Research Institute (WCHRI) at the University of Alberta along with two other studies also recruiting pregnant women. Upon having pregnancy confirmed at a medical clinic, potential participants were asked if they would be interested in participating in pregnancy research. If the individual was interested, her name and telephone number was forwarded to the WCHRI office. Names were randomly assigned between the three studies. Once names and telephone numbers were received from WCHRI, potential participants were called to invite the woman to participate in the study. It was made clear to participants that they would be taking part in a pilot study to test questionnaires for a larger study. Pregnant women in the present study were not APrON participants. 

Non-pregnant participants were recruited through a variety of other methods as there was no affiliation with medical clinics to recruit these women. These methods included recruitment tables, posters, advertisements in newsletters and word-of-mouth at a variety of locations including the University of Alberta community and City of Edmonton recreation facilities.

This research was approved by the Health Research Ethics Board at the University of Alberta, Human Research Ethics Study Number Pro00003163. All participants provided informed consent prior to participation.

### 2.2. Demographic Assessment

All participants reported age, height, marital status, parity, ethnicity, chronic illness, education level, employment status, and annual household income. Pregnant participants reported their weight immediately prior to pregnancy, and number of week’s gestation. Non-pregnant participants reported their current weight. Body mass index (BMI) was calculated by dividing weight (in kilograms) by height (in metres) squared.

### 2.3. The Food Frequency Questionnaire

The FFQ was adapted from the Canadian version of the Diet History Questionnaire (DHQ) [16] which was originally developed by the National Cancer Institute in the United States [17]. The original DHQ utilized the United States Department of Agriculture (USDA) Nutrient Database for Standard Reference (SR11) [17]. Csizmadi *et al.* [16] adapted the FFQ for use in Canada to reflect differences in Canadian nutrient fortification practices and food availability. The changes made utilized the Canadian Nutrient File 2007b [18] nutrient database for analyses. The FFQ was further adapted for the APrON study to address nutrients of concern prior to pregnancy [8] as well as incorporating culturally appropriate foods. Changes included the addition of foods fortified with omega-3 fatty acids (eggs, juice, margarine, milk, soymilk, and yogurt), an expanded fish section, and more multigrain/flax grain products. Additionally, some questions were re-ordered or combined. The nutrient profiles of additional foods were added using the Canadian Nutrient File 2007b [18].

The FFQ was a self-administered, semi-quantitative, retrospective questionnaire that asked about dietary intake over the 12 months prior to pregnancy for pregnant women or the past 12 months for non-pregnant women. Since women were recruited over an 18 month time frame, and “past 12 months” was used as the time over which diet was reported, differences that occur in dietary intake according to seasonality were minimized [15].

### 2.4. Comparison between Groups

Pregnant and non-pregnant women both completed the same FFQ. All data reported were for food and beverages only, supplements were not included.

Nutrient intakes in pregnant women were compared to nutrient intakes in non-pregnant women. The key nutrients selected for comparison were long-chain omega-3 fatty acids, folate, vitamin B_6_, vitamin B_12_, calcium, vitamin D and iron. In addition, total energy and macronutrient intake (carbohydrate, protein, and fat) were compared between groups.

### 2.5. Data Entry

Responses to all FFQs were double-entered into a key-punch program utilizing Microsoft Excel^®^ (Excel version 2007) to ensure accuracy.

### 2.6. Statistical Analysis

Assessment for outliers for FFQ data was completed based on unrealistic reported energy intakes of <600 kcal or >3500 kcal per day, as recommended by Csizmadi *et al.* [16]. Independent samples *t*-tests were used to compare differences between groups for continuous demographic variables while chi square analysis and Fisher’s exact test were used to examine the differences in categorical demographics between groups. Independent samples *t*-tests were used to compare mean intake between groups of the key nutrients measured by the FFQ. 

Analyses were performed using SPSS (version 18.0, SPSS Inc, Chicago, IL, USA) except for demographic variables: parity, education level, household income and BMI classification where STATA (version 10, StataCorp LP, College Station, TX, USA) was used.

## 3. Results

### 3.1. Participant Information

Overall, 98 pregnant women and 103 non-pregnant women completed the study. Nine participants (seven pregnant and two non-pregnant) were excluded from all analyses on the basis of unrealistic energy intake reported on the FFQ [16]. Participant demographic information is described in Table 1. Compared to the non-pregnant women, the women in the pregnant group were more likely to have a higher pre-pregnancy BMI (*p* = 0.003), be married (*p* < 0.001), have more children (*p* = 0.005), be employed (*p* = 0.025), and have a higher household income (*p* < 0.001). There were no significant differences found between groups for age, ethnicity, chronic illness, or level of education.

### 3.2. FFQ Nutrient Intake Comparison between Groups

Energy, macronutrient, and key micronutrient intakes are shown in Table 2. The pregnant women reported a significantly higher mean intake of saturated fat (SFA) (*p* < 0.05), *trans* fat (*p* < 0.01), and calcium (*p* < 0.05) compared to the non-pregnant group. The non-pregnant women reported a significantly higher mean intake of alcohol (*p* < 0.05). Intakes of energy and all other macronutrients and key micronutrients were similar between groups.

**Table 1:** Participant Demographic Characteristics.

	Pregnant (*n* = 91)	Non-Pregnant (*n* = 101)	*p* value
	**Mean (SD) ^a^**		
Age (years)	30.8 ± 5.0	29.1 ± 7.7	0.072
Weeks Gestation	31.0 ± 8.2	N/A	N/A
Body Mass Index (BMI) (kg/m^2^)	25.1 ± 6.7	22.8 ± 3.1	0.003 **
	**(%) ^b^**		
**Marital Status**			<0.001 **
Married/Common Law	97%	51%	
Single/Divorced/Other	3%	49%	
**Parity**			0.005 *
0	53%	68%	
1	32%	13%	
2 or more	15%	19%	
**Ethnicity**			0.293
Caucasian	77%	83%	
Other	23%	17%	
**Chronic Illness**			0.620
No	80%	83%	
Yes	20%	17%	
**Education Level**			0.616
High school graduate or less	7%	4%	
Some college or university	16%	20%	
College or university degree or above	77%	76%	
**Employment Status**			0.025 *
Employed/Self-Employed	70%	55%	
Unemployed ^c^	30%	45%	
**Annual Household Income**			<0.001 **
<$30,000	3%	35%	
$30,000–59,000	18%	23%	
>$60,000	79%	42%	

* *p*-Value significant at *p* < 0.05 level; ** *p*-value significant at *p* < 0.01 level; **^a^** *t*-test for independent samples; **^b^** Chi square analysis (marital status, ethnicity, chronic illness, employment status) or Fisher’s Exact Test (parity, education, income, BMI) as appropriate; **^c^** Included students, homemakers, unemployed or other. Abbreviations: BMI (body mass index), kg (kilograms), m (metres), SD (standard deviation).

**Table 2:** Comparison between Groups of Energy and Nutrient Intake Measured by FFQ.

Nutrient	Pregnant ^a^ (*n* = 91) (mean ± SD)	Non-Pregnant ^b^ (*n* = 101) (mean ± SD)	*p*-value
Energy (kcal)	1927 ± 537	1869 ± 529	0.456
Carbohydrate (g)	261.8 ± 82.2	246.9 ± 78.9	0.204
Fibre (g)	22.2 ± 9.2	24.1 ± 10.2	0.169
Protein (g)	77.6 ± 25.6	77.3 ± 26.2	0.958
Fat (g)	67.6 ± 22.1	66.1 ± 25.2	0.660
Saturated Fat (g)	22.1 ± 8.6	19.6 ± 8.1	0.039 *
MUFA (g)	26.6 ± 9.8	27.1 ± 12.0	0.733
PUFA (g)	13.1 ± 4.7	13.5 ± 5.8	0.527
ALA (g)	1.8 ± 0.8	1.7 ± 0.7	0.265
EPA/DHA (g)	0.14 ± 0.13	0.17 ± 0.21	0.246
*Trans* Fat (g)	3.5 ± 1.4	2.9 ± 1.4	0.007 **
Cholesterol (mg)	200.6 ± 75.6	182.7 ± 82.7	0.120
Alcohol (g)	3.7 ± 4.9	5.6 ± 7.3	0.043 *
Folate (µg)	369 ± 124	392 ± 148	0.250
Vitamin B_6_ (mg)	2.1 ± 0.8	2.1 ± 0.8	0.856
Vitamin B_12_ (µg)	5.1 ± 2.3	5.0 ± 2.3	0.802
Calcium (mg)	1146 ± 556	988 ± 408	0.026 *
Vitamin D (µg)	5.8 ± 3.5	5.2 ± 3.2	0.204
Iron (mg)	15.3 ± 5.0	16.4 ± 6.0	0.193

* Independent samples *t*-test significant at *p* < 0.05 level; ** Independent samples *t*-test significant at *p* < 0.01 level. **^a^** Energy and macronutrient intake for the year prior to becoming pregnant; **^b^** Energy and macronutrient intake for the past year. Abbreviations: FFQ (food frequency questionnaire), kcal (kilocalories), g (grams), mg (milligrams), µg (micrograms), SD (standard deviation), MUFA (monounsaturated fat), PUFA (polyunsaturated fat), ALA (alpha-linolenic acid), EPA/DHA (Eicosapentaenoic acid/Docosahexaenoic acid).

## 4. Discussion

A novel comparison between groups was used in this study to approach the question of whether or not food intake measured by the FFQ was similar between pregnant women reporting intake in the year prior to pregnancy and non-pregnant women reporting intake for the past year. It appeared that there were some differences between groups in dietary intake, however, it is not known whether these differences were due to actual differences in food intake between groups or differences in recall bias between groups. It is not a traditional assessment of the relative validity of a dietary intake tool whereby a tool is compared to a gold standard or secondary method. Assessment of relative validity in this population is difficult. Many studies have examined the relationship between FFQs and alternate methods of diet assessment utilizing a variety of populations, sample sizes, FFQs, reference time periods, and reference diet assessment methods [19]. In general, FFQs are typically believed to overestimate nutrient intake although it may be that some overestimate intake more than others or even underestimate intake [13,20,21].

Overall, the FFQ provided a similar estimate of energy, macronutrients and key micronutrients: folate, vitamin B_6_, vitamin B_12_, vitamin D and iron between groups. However, saturated fat, *trans* fat, and calcium intakes were reported as significantly higher, and alcohol intake was reported as significantly lower in the pregnant group recalling the year prior to pregnancy compared to the non-pregnant group recalling the past year. Reasons for these differences could be multi-fold but cannot be completely discerned in this study. It is possible that: (1) if planning a pregnancy, some women may have made dietary changes in the year prior to becoming pregnant. For example, a woman planning a pregnancy may decrease or eliminate intake of alcoholic beverages and increase consumption of other beverages, such as milk. The mean difference in calcium intake between groups was 158 mg which is approximately the amount of calcium in half a cup of milk [18]. Saturated and *trans* fats may also be present in milk although in varying amounts depending on the type of milk. Thus even if some of the pregnant women changed their beverage consumption as a result of planning pregnancy, intake of these nutrients may have been higher. (2) There may have been increased recall bias among the pregnant women such that they perceived a “healthier” diet in the year prior to pregnancy. Some research supports the idea that women intending pregnancy make positive behaviour changes including higher levels of physical activity and lower smoking rates compared with non-pregnant women [22], lower alcohol intake [23] and taking a multivitamin [24] and/or folic acid supplement [22,23,25]. In contrast, other pre-conception research indicates that women who are planning a pregnancy do not differ greatly from other non-pregnant women with respect to health behaviours such as alcohol use [22,24,25], smoking [23,25], fruit and vegetable consumption [23,25] and physical activity [25]. There may have been differences between groups in usual intake irrespective of whether the pregnancy was planned or not. In the present study, data on whether the pregnancy was planned was not collected so a definitive statement on this issue cannot be made.

Pre-pregnancy dietary intakes in this study were somewhat similar to pre-pregnancy intake reported by pregnant women in Portugal [26] and the Netherlands [13,20]. Women in the present study were observed to have lower energy intake [20,26], lower fat intake [13], higher vitamin D and lower vitamin B_12_ [26]. However, these studies used different FFQs, examined women who were planning a pregnancy or not [20], and were administered at different time points, such as during the first trimester [26] or after delivery [13], which makes direct comparison difficult. 

The primary limitation of this study was that neither FFQs nor any other diet assessment tool is considered a gold standard. A FFQ was chosen because it was the only tool that could retrospectively assess nutrient intake prior to pregnancy. The reality of recruitment procedures for the APrON study as well as the fact that many pregnancies are unplanned [27] limited the method to retrospective assessment. In addition to retrospective assessment, there are other times when FFQs are the appropriate choice for diet assessment. For example, a FFQ may more accurately measure nutrients with sporadic intake as opposed to daily intake. In this case a 24 h recall may miss intake of these nutrients. As such, long-chain omega-3 fatty acid intake may be best measured by FFQ as foods containing high levels of long-chain omega-3 fatty acids (*i.e.*, fatty fish) are typically not eaten on a daily basis in Canada [28].

Another limitation was that the pregnant women were recalling a 12 month period that was approximately seven to eight months prior (*i.e.*, before they were pregnant) as opposed to the non-pregnant women who were simply recalling the past 12 months. In addition, there was variation in the point of gestation of the pregnant women which resulted in some women having to recall a period that was longer than others. The extent to which habitual dietary recall is affected by these differences in time to recall is difficult to quantify. The fact that a FFQ is meant to capture habitual dietary intake may lessen the impact of these differences in recall period.

One of the strengths of the study, was that recruitment took place over approximately 18 months which would minimize the effect of seasonality on reporting food intake. Additionally, in terms of the FFQ, was that every effort was made to update the food list to include new fortified foods as well as an improved variety of foods. However, a weakness of FFQs in general is that they continually need updating to reflect changes in the food supply.

In addition, there were significant differences in the demographic characteristics of the two groups. This may have augmented the differences observed when comparing the FFQ between groups. For example, the significantly lower BMI of non-pregnant women compared to pregnant women may indicate differences in dietary intake patterns between groups.

Future research on this tool should include comparison against a reference tool with multiple days of dietary intake in order to generate stronger conclusions regarding the relative validity of the FFQ. In the future, it would also be important to collect whether the pregnancy was planned or not.

## 5. Conclusions

This is the first time to the researchers’ knowledge, that the assessment of a pre-pregnancy dietary assessment tool has been completed. The FFQ has a role in dietary assessment of the pre-pregnancy time period as it is a critical period but is difficult to assess.

The FFQ provided a similar estimate of dietary intake between pre-pregnant and non-pregnant groups for energy, macronutrients, and key micronutrients long-chain omega-3 fatty acids, folate, vitamin B_6_, vitamin B_12_, vitamin D and iron. With the data collected in the current study, it is not possible to state that the FFQ is relatively valid. However, there is also insufficient evidence to state that the FFQ is invalid. The FFQ is currently being used in the Alberta Pregnancy Outcomes and Nutrition (APrON) cohort study. The APrON study sample size will be very large and it is expected that at the group level, the large sample size will assist with removing some of the variability in intake measured by the FFQ. However, considering the limitations of the tool and the populations recruited in this study, it is essential that data from this questionnaire be interpreted and utilized with caution.
